# Association of the Three Common SNPs of Cyclooxygenase-2 Gene (rs20417, rs689466, and rs5275) with the Susceptibility of Breast Cancer: An Updated Meta-Analysis Involving 34,590 Subjects

**DOI:** 10.1155/2014/484729

**Published:** 2014-08-18

**Authors:** Zhi-Jun Dai, Yong-Ping Shao, Xiao-Bin Ma, Dan Xu, Wei Tang, Hua-Feng Kang, Shuai Lin, Meng Wang, Hong-Tao Ren, Xi-Jing Wang

**Affiliations:** ^1^Department of Oncology, The Second Affiliated Hospital of Xi'an Jiaotong University, Xi'an 710004, China; ^2^Center for Translational Medicine, Frontier Institute of Science and Technology (FIST), Xi'an Jiaotong University, Xi'an 710004, China; ^3^School of Chemistry and Chemical Engineering, Shaanxi Normal University, Xi'an 710061, China

## Abstract

Several single nucleotide polymorphisms have been identified in cyclooxygenase-2 (COX-2) genes (e.g., −765 G>C (rs20417), −1195G>A (rs689466), and 8473 C>T (rs5275)). The association of these SNPs with the risk of different cancer types is still controversial. This study aims to evaluate the correlation between these SNPs and breast cancer risk in different ethnic groups. We have searched PubMed, Web of Knowledge, and Embase for relevant studies. Odds ratios (ORs) with 95% confidence intervals (CIs) were used to estimate the strength of the associations. A total of 13 studies (15,330 cases and 19,260 controls) were eligible for meta-analysis. This meta-analysis showed that COX-2 rs20417 polymorphism was correlated with an increased risk of breast cancer in Caucasians, while rs689466 was associated with a decreased risk of breast cancer in Caucasians. The rs5275 polymorphism had no association with breast cancer risk.

## 1. Introduction

Breast cancer is the most common cancer in women worldwide [1]. It is a multifactorial disease caused by complex genetic and environmental factors [2]. Allele variants in oncogenes are candidate genetic risk factors that may alter breast cancer onset and outcome. Previous researches have suggested that the risk of breast cancer is affected by multiple environmental factors as well as genetic alterations, such as genetic polymorphisms [3, 4].

Cyclooxygenase (COX), also known as prostaglandin endoperoxide synthetase (PTGS), plays an important role in the inflammatory process by converting arachidonic acid to prostaglandins (PG) [5]. There are two COX isoforms: COX-1 and COX-2. COX-1 is present in many tissues and is involved in PG synthesis. By contrast, COX-2 is not detected in most normal tissues but is often overexpressed in many tumor types [6]. COX-2 can be rapidly induced by a variety of mitogenic and inflammatory stimuli and elevate the production of prostaglandins, which contribute to tumor occurrence and progression by modulating cell proliferation, apoptosis, and angiogenesis [6–8]. In breast cancer, several studies have suggested that moderate to high COX-2 expression is related to the genesis of mammary tumors and the expression level is associated with the aggressiveness of breast cancer, including large tumor size, positive axillary lymph node metastases, and HER2-positive tumor status [9–11]. Targeted inhibition of COX-2 blocked the proliferation of breast cancer cell lines* in vitro* and prevented the occurrence of rat breast cancer chemically induced by DMBA [12].

Genetic polymorphisms in COX-2 have been shown to alter its expression and influence the susceptibility to various carcinomas [13, 14], including breast cancer [15]. The human COX-2 gene (also known as PTGS2) is located on chromosome 1q25.2-q25.3 and consists of 10 exons spanning 8.3 kb [16]. Several single-nucleotide polymorphisms (SNPs) in COX-2 have been identified, of which three functional SNPs, −765 G>C (rs20417), −1195G>A (rs689466) in the promoter region, and the 8473 C>T (rs5275) in the 3′UTR of COX-2, have been widely investigated [13–15].

Previous functional studies have suggested that the rs20417 polymorphism may eliminate a Sp1-binding site but create an E2F binding site and result in altered COX-2 expression [13]. The rs5275 polymorphism was shown to be associated with the alteration of mRNA level of the gene as sequences within the 3′UTR are important for message stability and translational efficiency [17]. There are many studies that have investigated the association between COX-2 polymorphisms and breast cancer risk. However, the results are inconsistent. For example, Fawzy et al. reported that rs5275 polymorphism was associated with the BC in Egyptian women. The individuals with rs5275 CC genotypes showed significant increase in plasma PGE2 levels [18]. However, Brasky et al. demonstrated that rs5275 had no association with breast cancer risk in Caucasians [19]. In our previous study, variant genotypes of COX-2 rs20417 G>C (GC/CC) were associated with increased breast cancer risk. Furthermore, the increased risk was more prominent among the younger subjects (OR = 1.61, 95% CI = 1.00–2.61). The variant genotypes were also associated with tumor size (OR = 3.01, 95% CI = 1.47–6.12) [20].

To clarify the role of COX-2 polymorphisms in breast cancer risk, Yu et al. conducted a meta-analysis on the associations between several COX-2 polymorphisms and breast cancer risk. The results suggested borderline increased risk of breast cancer with rs5277 but no significant associations with the rs20417 and rs5275 polymorphisms [15]. However, of the studies included in their meta-analysis, only two studies were carried out in Asians [23, 28] and the rs689466 polymorphism was not involved. To make a more precise estimation, we conduct the present meta-analysis on all eligible case-control studies to evaluate the association between the three common SNPs (rs20417, rs689466, and rs5275) and breast cancer susceptibility.

## 2. Materials and Methods

### 2.1. Publication Search

We searched the electronic databases of PubMed, Web of Knowledge, and Embase to collect articles with case-control studies related to the association of COX-2 polymorphisms with breast cancer risk. The keywords were as follows: breast cancer/breast carcinoma, Cyclooxygenase-2/COX-2/PTGS, and polymorphism/genotype/SNP. All qualified studies prior to February 28, 2014, were included. The eligible literature must be published in English. Furthermore, reference lists of main reports and review articles were also reviewed manually to identify additional relevant publications.

### 2.2. Selection Criteria

The following criteria were used to select studies for further meta-analysis: (1) case-control studies; (2) studies that evaluated the associations between COX-2 polymorphisms and breast cancer risk; (3) studies that contained at least two comparison groups (cancer versus control); (4) studies that included detailed genotyping data.

### 2.3. Data Extraction and Synthesis

Articles were reviewed independently by two reviewers and data with discrepancies were discussed by all authors. For each included study, the following information was collected: first author, year of publication, country of origin, ethnicity, source of control, total number of cases and controls, and genotyping methods as well as number of cases and controls with the different genotypes. Different ethnic groups were categorized as Caucasian, Asian, African, and “mixed.” All the case and control groups were well controlled. The noncancer controls had no history of gynecologic disease, and there was no present evidence of any malignant disease. The histories of chronic inflammatory condition or other malignancies of the patients were not considered in this study.

### 2.4. Statistical Analysis

The associations between COX-2 polymorphisms and breast cancer risk were measured by odds ratio (OR) with 95% confidence interval (CI). The significance of the pooled OR was determined by the *Z* test.

The meta-analysis assessed association by using 4 different genetic models: (1) dominant genetic model—the comparison groups were the wild-type homozygous genotype versus the variant allele-positive genotypes (AA + Aa versus aa); (2) recessive genetic model—the comparison groups were the variant homozygous genotype versus the rest (AA versus aa + Aa); (3) homozygous genetic model—comparison was between the 2 homozygous genotypes (AA versus aa); and (4) allele contrast genetic model—the comparison was between the heterozygous and the homozygous wild-type genotype groups (Aa versus aa (where “a” is the wild-type allele and “A” is the variant allele)).

Statistical heterogeneity among studies was assessed with the *Q* and *I* ^2^ statistics. If the *P* value of heterogeneity test was more than 0.1 (*P* ≥ 0.1), the pooled OR estimate of the study was calculated by the fixed-effects model. Otherwise, the random-effects model was used [11]. The value of the *I* index is used to assess the degree of heterogeneity (*I* ^2^ < 25%: no heterogeneity; 25% < *I* ^2^ < 50%: moderate heterogeneity; 50% < *I* ^2^ < 75%: high heterogeneity; *I* ^2^ > 75%: extreme high heterogeneity). Publication bias was evaluated by the funnel plot and further assessed by the method of Egger's linear regression test. All statistical analyses were carried out with the review manager version 5.1 (Revman; The Cochrane Collaboration, Oxford, UK).

## 3. Results

### 3.1. Characteristics of Studies

**Figure 1:**
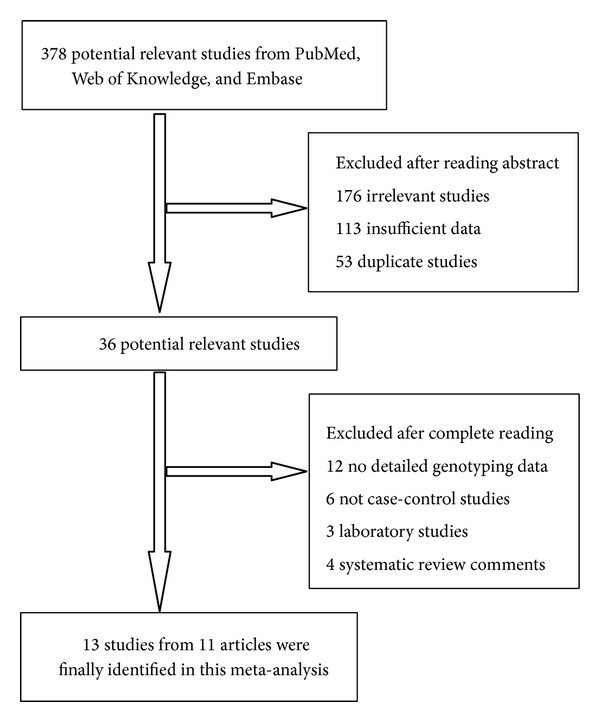
Flow chart of studies selection in this meta-analysis.

As shown in Figure 1, a total of 378 potential publications were initially extracted. After reading the abstracts, we excluded 176 irrelevant studies, 113 studies with insufficient data, and 53 duplicated ones. In-depth reading of the remaining articles led to further exclusion of 12 articles with no detailed genotyping data, 6 studies with no case-control, 3 laboratory studies, and 4 systematic review articles. Finally, 13 studies from 11 articles were collected for this meta-analysis.

Overall, 13 studies on COX-2 polymorphisms and breast cancer risk were identified [16, 18–27], including a total of 15,330 cases and 19,260 case-free controls. The characteristics of the included studies are listed in Table 1. Among the eligible 13 studies, nine studies were carried out in Caucasians from USA, Austria, Denmark, Brazil, and nine European countries. Two were based on Asian background and were carried out in China. Only one study carried out in Egypt was based on African background. One study was on mixed ethnic groups. All studies were case-controlled. All breast cancers were confirmed by histology or pathology. Moreover, controls were mainly matched by age. Five studies were hospital-based and eight were population-based.

### 3.2. Meta-Analysis Results

As shown in Table 2, the frequencies of the minor allele varied widely across the eligible studies, ranging from 0.06 to 0.28 (rs20417), 0.12 to 0.54 (rs689466), and 0.18 to 0.45 (rs5275). The average frequencies of the minor allele in the three polymorphisms were 0.17, 0.22, and 0.33, respectively.

**Figure 2:**
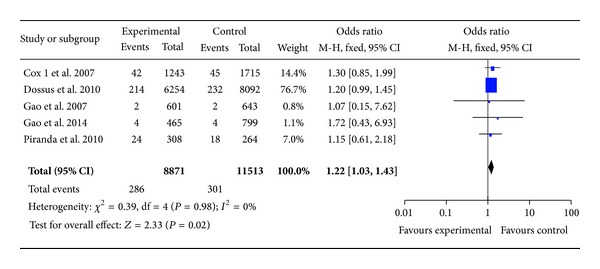
. Forest plots of COX-2 rs20417 polymorphism and breast cancer risk in the overall population (CC versus GG + GC). The squares and horizontal lines correspond to the study specific OR and 95% CI. The area of the squares reflects the weight (inverse of the variance). The diamond represents the summary OR and 95% CI.

The main results of this meta-analysis were listed in Table 3. There were 6 studies with 9,938 cases and 12,618 controls for rs20417. As shown in Table 3 and Figure 2, rs20417 polymorphism has association with breast cancer risk in the overall population based on homozygote comparison (CC versus GG: OR = 1.21, 95% CI = 1.02–1.42, *P* = 0.03) and the recessive model (CC versus GG + GC: OR = 1.22, 95% CI = 1.03–1.43, *P* = 0.02). However, there are no significant associations in other genetic models (C versus G: OR = 1.04, 95% CI = 0.98–1.10, *P* = 0.17; heterozygote comparison (GC versus GG): OR = 0.97, 95% CI = 0.91–1.03, *P* = 0.35; dominant model (GC + CC versus GG): OR = 1.01, 95% CI = 0.96–1.08, *P* = 0.64). In the stratified analysis by ethnicity, the effects remained in Caucasians (homozygote comparison: OR = 1.20, 95% CI = 1.02–1.42, *P* = 0.03; recessive model: OR = 1.21, 95% CI = 1.03–1.43, *P* = 0.02), but not in Asians (Table 3).

**Figure 3:**
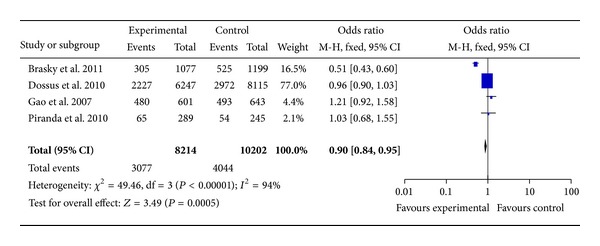
Forest plots of COX-2 rs689466 polymorphism and breast cancer risk in the overall population (AA + AG versus GG). The squares and horizontal lines correspond to the study specific OR and 95% CI. The area of the squares reflects the weight (inverse of the variance). The diamond represents the summary OR and 95% CI.

There were 4 studies with 8,214 cases and 10,202 controls for assessing the relationship between COX-2 rs689466 polymorphism and breast cancer susceptibility. As shown in Table 3 and Figure 3, there was no association in these four genotypes (A versus G: OR = 0.99, 95% CI = 0.94–1.04, *P* = 0.69; homozygote comparison (AA versus GG): OR = 1.01, 95% CI = 0.88–1.15, *P* = 0.93; heterozygote comparison (AG versus GG): OR = 0.98, 95% CI = 0.92–1.05, *P* = 0.59; recessive model (AA versus GG + AG): OR = 1.01, 95% CI = 0.89–1.15, *P* = 0.85). However, rs689466 polymorphism has association with breast cancer risk based on the recessive model (AA + AG versus GG: OR = 0.90, 95% CI = 0.87–0.95, *P* = 0.002). In the stratified analysis, when analyzed by the dominant model, the OR was 0.88 (95% CI = 0.83–0.94) (*P* < 0.0001) among Caucasians. These results suggested that the individuals with AA or AG alleles have a 12% decreased risk of breast cancer compared with those with GG allele in Caucasians.

**Figure 4:**
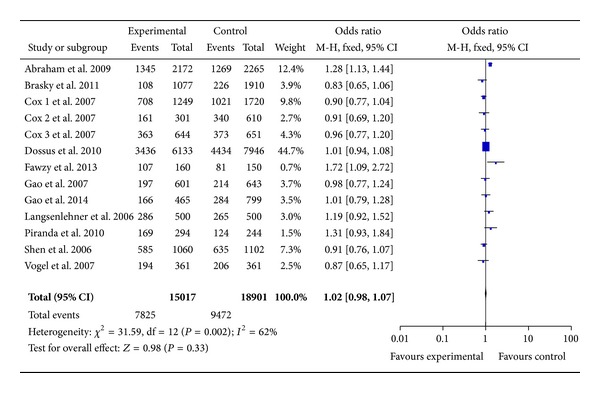
Forest plots of COX-2 rs5275 polymorphism and breast cancer risk in the overall population (TT + TC versus CC). The squares and horizontal lines correspond to the study specific OR and 95% CI. The area of the squares reflects the weight (inverse of the variance). The diamond represents the summary OR and 95% CI.

13 studies with 15,017 cases and 18,901 controls were used to evaluate the relationship between COX-2 rs5275 polymorphism with breast cancer risk. As shown in Table 3 and Figure 4, there was no significant association in rs5275 polymorphism (homozygote comparison: OR = 1.04, 95% CI = 0.96–1.12, *P* = 0.34; heterozygote comparison: OR = 0.99, 95% CI = 0.95–1.04, *P* = 0.81; dominant model: OR = 1.02, 95% CI = 0.98–1.07, *P* = 0.33, and recessive model: OR = 1.04, 95% CI = 0.97–1.12, *P* = 0.27). When stratified by ethnicity, there was also no association between rs5275 and breast cancer risk in both Caucasians and Asians (Table 3).

### 3.3. Publication Bias

**Figure 5:**
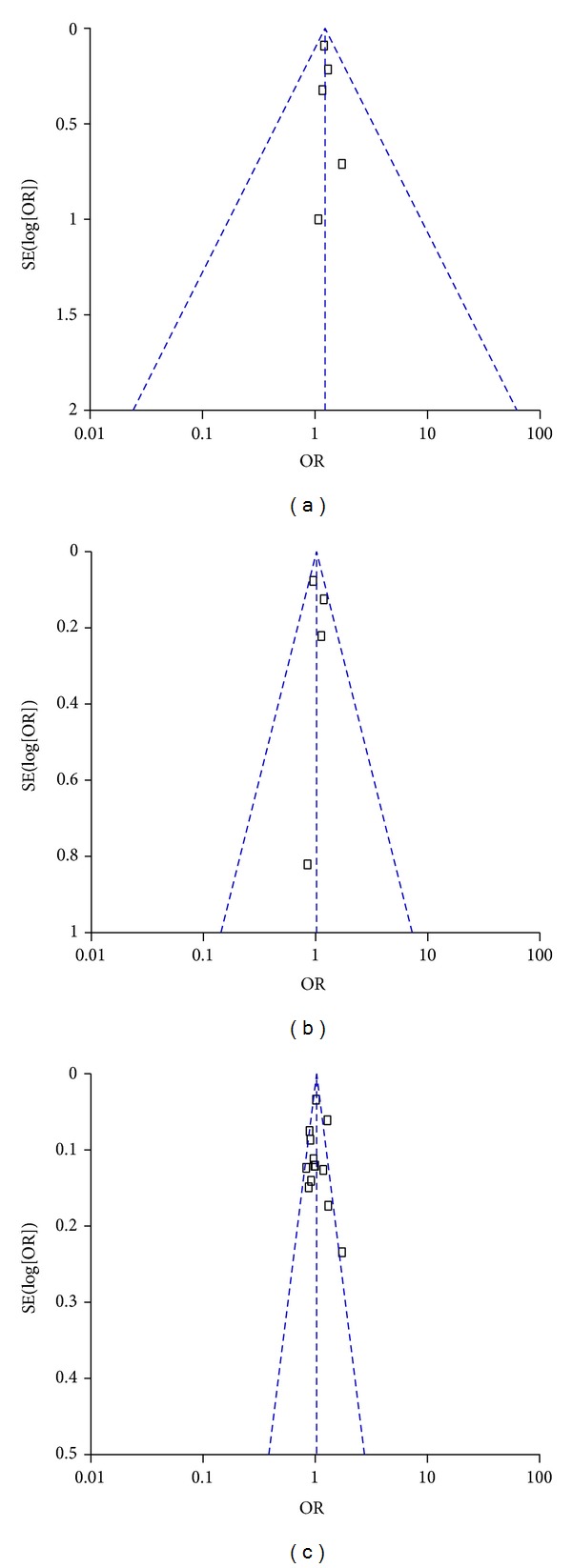
Funnel plot assessing evidence of publication bias from the eligible studies. (a) −765 G>C (rs20417); (b) −1195G>A (rs689466); and (c) 8473 C>T (rs5275).

In this meta-analysis, we performed funnel plot and Egger's test to access the publication bias. As shown in Figure 5, the funnel plots failed to detect any obvious asymmetry in all genotypes in overall population, and the results of Egger's test revealed no publication bias (*P* > 0.05). Therefore, no significant publication bias was found in this meta-analysis.

## 4. Discussion

The present meta-analysis, including 15,330 cases and 19,260 controls from 13 case-control studies, was conducted to evaluate the association between the three common SNPs [−765 G>C (rs20417), −1195 G>A (rs689466), and 8473 C>T (rs5275)] in the COX-2 gene and breast cancer risk.

A previous study by Yu et al. [15] failed to detect an association between rs20417 and breast cancer risk. There were only three studies with 2,901 cases and 3,463 controls for rs20417 in Yu's meta-analysis [15]. In this study, there were six studies with 9,938 cases and 12,618 controls included to evaluate the relationship between rs20417 polymorphism and breast cancer risk. The results showed that rs20417 polymorphism was associated with breast cancer risk in the overall population based on homozygote comparison (CC versus GG: OR = 1.21, 95% CI = 1.02–1.42, *P* = 0.03). Moreover, in a stratified analysis by ethnicity using the recessive model, we found that the association remained in Caucasians (homozygote comparison: OR = 1.20, 95% CI = 1.02–1.42, *P* = 0.03; recessive model: OR = 1.21, 95% CI = 1.03–1.43, *P* = 0.02), but not in Asians. These results suggest ethnic differences in genetic backgrounds and the environment in which they live play a possible role in breast carcinogenesis [29].

In Zhu et al.'s meta-analysis [30], they showed that individuals with the rs20417 were associated with higher cancer risk than those with the −765GG genotype. Stratified analysis further revealed that this effect was maintained in colorectal carcinoma and esophageal cancer in Asian descents. However, the rs5275 polymorphism was not associated with cancer risk although in breast and lung cancer this allele was correlated with decreased risk.

In the present meta-analysis, 13 studies with 15,017 cases and 18,901 controls concerning the rs5275 polymorphism were included. We found no significant association of rs5275 polymorphism with breast cancer risk (homozygote comparison: OR = 1.04, 95% CI = 0.96–1.12, *P* = 0.34; heterozygote comparison: OR = 0.99, 95% CI = 0.95–1.04, *P* = 0.81; dominant model: OR = 1.02, 95% CI = 0.98–1.07, *P* = 0.33, and recessive model: OR = 1.04, 95% CI = 0.97–1.12, *P* = 0.27). When stratified by ethnicity, similar results were observed in both Caucasians and Asians.

In the previous meta-analysis by Tang et al. [14], there was an association of the rs689466 polymorphism with cancer risk in Asian populations but not in Europeans. Our results indicate that rs689466 polymorphism has association with breast cancer risk based on the recessive model (AA + AG versus GG: OR = 0.90, 95% CI = 0.87–0.95, *P* = 0.002). In the stratified analysis, when analyzed by the dominant model, the OR was 0.88 (95% CI = 0.83–0.94) (*P* < 0.0001) among Caucasians.

Some limitations of this meta-analysis should be noted. Firstly, this meta-analysis was based on pooled data and no individual data was available; thus, we could not assess the risk of cancer according to stratification by age, environment factors, and other risk factors of breast cancer. Secondly, most of the included studies did not investigate the chronic inflammatory condition and the history of taking nonsteroidal anti-inflammatory drugs. Thirdly, the included studies are mainly based on Caucasian background. There were only two studies based on Asian background and one based on African background. Larger scale multicenter studies based on Asians or Africans are warranted to further validate the association between COX-2 polymorphisms and breast cancer risk.

## 5. Conclusion

In summary, this meta-analysis points to the COX-2 rs20417 C allele as a risk factor for breast cancer among Caucasian subjects. On the contrary, the rs689466 allele has a decreased risk of breast cancer in Caucasians. The rs5275 C status does not seem capable of predicting breast cancer risk in both Caucasians and Asians.

## Figures and Tables

**Table 1:** Characteristics of the studies included in the meta-analysis.

First author	Year	Country	Ethnicity	Study design	Genotyping method	Source of control	Total sample size (case/control)	SNP number
Gao [20]	2014	China	Asian	CC	TaqMan	Hospital	465/799	1, 3
Fawzy [18]	2013	Egypt	African	CC	PCR-RFLP	Hospital	160/150	3
Brasky [19]	2011	USA	Caucasian	CC	TaqMan	Population	1077/1910	2, 3
Piranda [16]	2010	Brazil	Caucasian	CC	TaqMan	Population	318/273	1, 2, 3
Dossus [21]	2010	USA, Europe	Caucasian	CC	Illumina	Population	6292/8135	1, 2, 3
Abraham [22]	2009	EPIC	Caucasian	CC	TaqMan	Population	2200/2280	3
Gao [23]	2007	China	Asian	CC	PCR-RFLP	Hospital	601/643	1, 2, 3
Cox 1 [24]	2007	USA	Caucasian	CC	TaqMan	Population	1270/1762	1, 3
Cox 2 [24]	2007	USA	Caucasian	CC	TaqMan	Population	317/634	3
Cox 3 [24]	2007	USA	Caucasian	CC	TaqMan	Population	702/703	3
Vogel [25]	2007	Denmark	Caucasian	CC	TaqMan	Hospital	361/361	3
Langsenlehner [26]	2006	Austria	Caucasian	CC	TaqMan	Hospital	500/500	3
Shen [27]	2006	USA	Mixed	CC	PCR-RFLP	Population	1067/1110	1, 3

CC: case-control; PCR: polymerase chain reaction; RFLP: restriction fragment length polymorphism. EPIC: European Prospective Investigation of Cancer (a prospective study of diet and cancer being carried out in nine European countries). SNP: single-nucleotide polymorphisms; SNP number 1: −765G>C (rs20417); 2: −1195G>A (rs689466); 3: 8473T>C (rs5275).

**Table 2:** COX-2 polymorphisms genotype distribution and allele frequency in cases and controls.

First author	Genotype (*N*)								Allele frequency (*N*)				MAF
	Case				Control				Case		Control		
	Total	AA	AB	BB	Total	AA	AB	BB	A	B	A	B	
rs20417													
Gao 2014 [20]	465	394	67	4	799	719	76	4	855	75	1514	84	0.08
Piranda 2010 [16]	308	157	127	24	264	129	117	18	441	175	375	153	0.28
Dossus 2010 [21]	6254	4394	1646	214	8092	5694	2166	232	10434	2074	13554	2630	0.17
Gao 2007 [23]	601	526	73	2	643	582	59	2	1125	77	1223	63	0.06
Cox 1 2007 [24]	1243	865	336	42	1715	1185	485	45	2066	420	2855	575	0.17
Shen 2006 [27]	1067	670	387		1105	691	414		—	—	—	—	—
rs689466													
Brasky 2011 [19]	1077	660	271	34	1910	1199	471	54	1591	339	2869	579	0.18
Piranda 2010 [16]	289	224	62	3	245	190	51	3	510	68	431	57	0.12
Dossus 2010 [21]	6247	4020	1928	299	8115	5143	2562	410	9968	2526	12848	3382	0.20
Gao 2007 [23]	601	121	305	175	643	150	327	166	547	655	627	659	0.54
rs5275													
Gao 2014 [20]	465	299	132	34	799	515	244	40	730	200	1274	324	0.22
Fawzy 2013 [18]	160	53	71	36	150	69	67	14	177	143	205	95	0.45
Brasky 2011 [19]	1077	432	447	108	1910	732	782	226	1311	663	2246	1234	0.31
Piranda 2010 [16]	294	125	149	20	244	120	99	25	399	189	339	149	0.32
Dossus 2010 [21]	6133	2697	2664	772	7946	3512	3501	933	8058	4208	10525	5367	0.34
Abraham 2009 [22]	2172	927	985	260	2265	996	1010	259	2839	1505	3002	1528	0.35
Gao 2007 [23]	601	404	179	18	643	429	194	20	987	215	1052	234	0.18
Cox 1 2007 [24]	1249	541	567	141	1720	699	808	213	1649	849	2206	1234	0.34
Cox 2 2007 [24]	301	140	131	30	610	270	259	81	411	191	799	421	0.32
Cox 3 2007 [24]	644	281	296	67	651	278	294	79	858	430	850	452	0.33
Vogel 2007 [25]	361	167	150	44	361	155	165	41	484	238	475	247	0.33
Langsenlehner 2006 [26]	500	214	224	62	500	234	232	33	652	348	700	298	0.35
Shen 2006 [27]	1060	475	585		1102	467	635		—	—	—	—	—

A represents the major allele; B represents the minor allele. MAF: minor allele frequencies.

**Table 3:** Meta-analysis of the association between COX-2 polymorphisms and breast cancer risk.

Comparisons	OR	95% CI	*P* value	Heterogeneity		Effects model
				*I* ^2^	*P* value	
B versus A						
rs20417	1.04	0.98–1.10	0.17	56%	0.06	R
Caucasian	1.02	0.96–1.08	0.50	0%	0.92	F
Asian	1.45	1.15–1.84	0.002	0%	0.47	F
rs689466	0.99	0.94–1.04	0.69	33%	0.21	F
Caucasian	0.97	0.92–1.03	0.34	0%	0.58	F
Asian	1.14	0.97–1.33	—	—	—	—
rs5275	1.01	0.98–1.05	0.50	56%	0.01	R
Caucasian	1.00	0.97–1.04	0.80	41%	0.09	R
Asian	1.03	0.89–1.19	0.70	0%	0.51	F
BB versus AA						
rs20417	1.21	1.02–1.42	**0.03**	0%	0.97	F
Caucasian	1.20	1.02–1.42	**0.03**	0%	0.92	F
Asian	1.54	0.49–4.78	0.46	0%	0.68	F
rs689466	1.01	0.88–1.15	0.93	22%	0.28	F
Caucasian	0.95	0.82–1.10	0.52	0%	0.69	F
Asian	1.31	0.95–1.80	—	—	—	—
rs5275	1.04	0.96–1.12	0.34	66%	0.0008	R
Caucasian	1.01	0.94–1.10	0.72	58%	0.01	R
Asian	1.26	0.85–1.85	0.25	7%	0.30	F
AB versus AA						
rs20417	0.97	0.91–1.03	0.35	93%	<0.00001	R
Caucasian	0.94	0.88–1.01	0.07	95%	<0.00001	R
Asian	**1.49**	**1.15–1.91**	**0.002**	0%	0.53	F
rs689466	0.98	0.92–1.05	0.59	0%	0.59	F
Caucasian	0.97	0.91–1.04	0.44	0%	0.74	F
Asian	1.16	0.87–1.54	—	—	—	—
rs5275	0.99	0.95–1.04	0.81	0%	0.56	F
Caucasian	0.99	0.95–1.04	0.81	0%	0.47	F
Asian	0.96	0.80–1.14	0.96	0%	0.78	F
AB + BB versus AA						
rs20417	1.01	0.96–1.08	0.64	54%	0.05	R
Caucasian	1.00	0.93–1.06	0.93	0%	0.84	F
Asian	1.49	1.16–1.91	0.002	0%	0.49	F
rs689466	**0.90**	**0.87–0.95**	**0.0005**	94%	<0.00001	R
Caucasian	**0.88**	**0.83–0.94**	**<0.0001**	96%	<0.00001	R
Asian	1.21	0.92–1.58	—	—	—	—
rs5275	1.02	0.98–1.07	0.33	62%	0.002	R
Caucasian	1.02	0.97–1.07	0.42	66%	0.002	R
Asian	0.99	0.84–1.17	0.92	0%	0.86	F
BB versus AA + AB						
rs20417	**1.22**	**1.03–1.43**	**0.02**	0%	0.98	F
Caucasian	**1.21**	**1.03–1.43**	**0.02**	0%	0.94	F
Asian	146	0.47–4.56	0.51	0%	0.70	F
rs689466	1.01	0.89–1.15	0.85	0%	0.48	F
Caucasian	0.96	0.83–1.11	0.59	0%	0.76	F
Asian	1.18	0.92–1.51	—	—	—	—
rs5275	1.04	0.97–1.12	0.27	65%	0.0009	R
Caucasian	1.02	0.95–1.10	0.60	60%	0.01	R
Asian	1.28	0.87–1.87	0.21	15%	0.28	F

A: represents the major allele; B: represents the minor allele; F: fixed-effects model; R: random-effects model.
